# Cold snare polypectomy for duodenal adenomas in familial adenomatous polyposis: a prospective international cohort study

**DOI:** 10.1055/a-2165-7436

**Published:** 2023-11-10

**Authors:** Arthur S. Aelvoet, John G. Karstensen, Barbara A.J. Bastiaansen, Monique E. van Leerdam, Francesc Balaguer, Michal Kaminski, Roel Hompes, Patrick M.M. Bossuyt, Luigi Ricciardiello, Andrew Latchford, Rodrigo Jover, Maria Daca-Alvarez, Maria Pellisé, Evelien Dekker, European FAP Consortium

**Affiliations:** 1 1234 Department of Gastroenterology and Hepatology, Amsterdam UMC location University of Amsterdam, Amsterdam, the Netherlands; 2 Cancer Center Amsterdam, Amsterdam, the Netherlands; 3 1234 Amsterdam Gastroenterology Endocrinology Metabolism, Amsterdam, the Netherlands; 4 53137 Gastrounit, Copenhagen University Hospital - Amager and Hvidovre, Danish Polyposis Registry, Hvidovre, Denmark; 5 4321 Department of Clinical Medicine, University of Copenhagen, Copenhagen, Denmark; 6 Department of Gastrointestinal Oncology, Netherlands Cancer Institute, Amsterdam, the Netherlands; 7 4501 Department of Gastroenterology and Hepatology, Leiden University Medical Center, Leiden, the Netherlands; 8 Department of Gastroenterology, Institut d'Investigacions Biomèdiques August Pi i Sunyer (IDIBAPS), Centro de Investigación Biomédica en Red de Enfermedades Hepáticas y Digestivas (CIBEREHD), University of Barcelona, Hospital Clínic de Barcelona, Barcelona, Spain; 9 Department of Oncological Gastroenterology and Department of Cancer Prevention, Maria Sklodowska-Curie National Research Institute of Oncology, Warsaw, Poland; 10 Department of Surgery, Amsterdam UMC location University of Amsterdam, Amsterdam, the Netherlands; 11 26066 Department of Epidemiology and Data Science, Amsterdam UMC location University of Amsterdam, Amsterdam, the Netherlands; 12 Policlinico di Sant'Orsola, IRCCS Azienda Ospedaliero-Universitaria di Bologna, Bologna, Italy; 13 9296 Department of Medical and Surgical Sciences, University of Bologna, Bologna, Italy; 14 Polyposis Registry, St Mark's Hospital, Harrow, United Kingdom; 15 4615 Department of Surgery and Cancer, Imperial College London, London, United Kingdom; 16 16802 Instituto de Investigación Biomédica ISABIAL, Universidad Miguel Hernández, Servicio de Medicina Digestiva, Hospital General Universitario de Alicante, Alicante, Spain

**Keywords:** Familial adenomatous polyposis, Duodenal polyposis, Endoscopic resection

## Abstract

**Background and study aims** In patients with familial adenomatous polyposis (FAP), endoscopic resection of duodenal adenomas is commonly performed to prevent cancer and prevent or defer duodenal surgery. However, based on studies using different resection techniques, adverse events (AEs) of polypectomy in the duodenum can be significant. We hypothesized that cold snare polypectomy (CSP) is a safe technique for duodenal adenomas in FAP and evaluated its outcomes in our centers.

**Patients and methods** We performed a prospective international cohort study including FAP patients who underwent CSP for one or more superficial non-ampullary duodenal adenomas of any size between 2020 and 2022. At that time, this technique was common practice in our centers for superficial duodenal adenomas. The primary outcome was the occurrence of intraprocedural and post-procedural AEs.

**Results** In total, 133 CSPs were performed in 39 patients with FAP (1–18 per session). Median adenoma size was 10 mm (interquartile range 8–15 mm), ranging from 5 to 40 mm; 27 adenomas were ≥20 mm (20%). Of the 133 polypectomies, 109 (82%) were performed after submucosal injection. Sixty-one adenomas (46%) were resected en bloc and 72 (54%) piecemeal. Macroscopic radical resection was achieved for 129 polypectomies (97%). Deep mural injury type II occurred in three polyps (2%) with no delayed perforation after prophylactic clipping. There were no clinically significant bleeds, perforations or other post-procedural AEs. Histopathology showed low-grade dysplasia in all 133 adenomas.

**Conclusions** CSP for (multiple) superficial non-ampullary duodenal adenomas in FAP seems feasible and safe. Long-term prospective research is needed to evaluate whether protocolized duodenal polypectomies prevent cancer and surgery.

## Introduction

Familial adenomatous polyposis (FAP) is an inherited disorder resulting in the development of numerous colorectal adenomas, requiring colectomy at a young age to prevent colorectal cancer 1 . Nearly all patients with FAP also develop adenomas in the duodenum. The life-time risk for duodenal cancer is 4% to 10% 2 3 4 5 , and this cancer accounts for one of the most common FAP-related causes of death 6 .

Conventionally, extensive duodenal polyposis is considered a criterion for prophylactic duodenectomy to prevent malignant transformation; however, treatment burden for duodenectomy is high with significant morbidity (Clavien-Dindo III/IV 16%-53%) and mortality (0%-2%) risks 7 8 . With the technical advance in endoscopic resection made over the last two decades, many centers with expertise in FAP have started to perform endoscopic polypectomy in the duodenum. In an attempt to either prevent duodenal cancer and prevent or defer the need for surgical duodenectomy, many centers with expertise in FAP have started to perform endoscopic polypectomies in the duodenum.

Several studies shown that endoscopic treatment of duodenal adenomas resulted in downstaging of duodenal polyposis graded by Spigelman stage 9 10 11 . And because the Spigelman stage is associated with duodenal cancer risk, performing polypectomies might indeed reduce this risk. In one study, 74% of patients were free from duodenal surgery at 89 months after polypectomy 9 . This potential advantage, however, should be weighed against the risk of complications of endoscopic treatment.

In the large bowel, current guidelines advice cold snare polypectomy (CSP) for non-pedunculated colorectal polyps < 10 mm and accumulating evidence suggests that CSP might also be effective and safe in 10- to 19-mm and even larger colorectal polyps 12 . Compared to cautery-based techniques, CSP is associated with a lower risk of delayed bleeding, perforation, and post-polypectomy syndrome and a shorter procedure time 13 14 15 16 , which is favorable in a condition such as FAP in which multiple polyps often are removed in one session.

Compared to the colon, the complication risk of polypectomy in the duodenum is higher, presumably due to the thinner wall and increased vascularity. In two retrospective studies in FAP that included different resection techniques, the risk of perforation after endoscopic polypectomy in the duodenum was 2% to 3% and the risk of delayed bleeding 13% to 20% 9 10 . All delayed bleedings in Roos et al. occurred after polypectomy of large adenomas, all >20 mm. This considerable risk of adverse events (AEs) indicates a need for careful selection of lesions that can be safely resected, as well as a discussion about the preferred resection technique for duodenal adenomas in FAP. A few studies assessed the safety of CSP for duodenal adenomas. In a study including 10 patients with FAP, 332 CSPs were performed, mainly on polyps <10 mm (97%), and no (serious) AEs occurred, except for one intra-procedural bleed managed with hemoclips 17 . The same authors confirmed the safety of CSP in a recent update including 2413 CSPs 11 . In the sporadic setting, studies have shown that complications after duodenal CSP are rare (delayed bleeding 0%–4%, perforation 0%–4%), including comparative studies showing that these risks are higher after hot snare polypectomy (HSP) (delayed bleeding 8.7%–16.8%, perforation 1.4%–5.9%) 18 19 20 21 22 . A recently published European guideline, however, still recommends HSP for duodenal adenomas >5 mm 23 .

We aimed to evaluate the safety of CSP for superficial non-ampullary duodenal adenomas in a consecutive series of patients with FAP in our expert centers.

## Patients and methods

### Study design and subjects

From 2020 on, centers that participate in the European FAP Consortium have collected findings of endoscopic surveillance of patients with FAP in a prospectively maintained database. One of the aims of this prospective database is to study the safety and efficacy of endoscopic resection techniques for duodenal adenomas. For this study, we evaluated all CSPs for non-ampullary duodenal adenomas performed between 2020 and 2022 during one or more sessions in patients with FAP. A diagnosis of FAP was defined as having a constitutional pathogenic variant of the *APC* gene and/or having more than 100 colorectal adenomas and a family history of FAP.

The study was approved by the institutional review boards of all participating hospitals. All included patients gave informed consent prior to data collection. The study was registered at ClinicalTrials.gov (NCT04677998).

### CSP procedure

An en bloc CSP followed by a piecemeal CSP in the same session in a patient with extensive duodenal polyposis.Video 1

In the centers of the European FAP Consortium, the aim of duodenal polypectomy, apart from cancer prevention, is to prevent endoscopically unmanageable disease in the future, thereby obviating the need for duodenal surgery. The indications for performing polypectomy of non-ampullary duodenal adenomas are: adenomas ≥10 mm and adenomas ≥5 mm when in total more than 20 duodenal adenomas are present. All superficial adenomas are resected using CSP, while more protruding lesions are resected using HSP. Duodenal polypectomies were performed under sedation (propofol or midazolam plus fentanyl) by endoscopists with expertise in FAP on dedicated endoscopy lists in tertiary referral centers. Patients on vitamin K antagonists or direct-acting anticoagulants temporarily discontinued these drugs. Single-agent antiplatelet use was continued or discontinued depending on local protocols. Gastroscopes, duodenoscopes or pediatric colonoscopes were used for the included procedures. CO _2_ insufflation was used.

The procedure included assessment of the duodenum and stomach after which the adenomas with an indication for polypectomy were removed during one or multiple procedures, depending on the number and complexity of the lesions. The location, morphology, and size of the adenoma was assessed by the endoscopist prior to polypectomy, with or without narrow band imaging at the discretion of the endoscopists. Whether the submucosa was injected to lift the lesion, with or without adrenaline, was also left to the discretion of the endoscopist. Submucosal lifting and CSP was referred to as cold endoscopic mucosal resection (EMR) in some previous studies 20 22 . Most lesions of 5 to 9 mm were resected en bloc with CSP and lesions ≥10 mm with piecemeal CSP. The post-polypectomy site was carefully inspected to rule out residual adenomatous tissue and to check for hemostasis and deep mural injury. Hemoclips were placed at the endoscopist’s discretion. Post-procedural clinical admission for observation was not routinely planned for all patients, but left to the discretion of the treating endoscopist and local protocols. Standard prescription of prophylactic proton pump inhibitors was not advised, but left to the discretion of the treating endoscopist. No standard post-procedural dietary restrictions were advised. Video 1 shows an en bloc CSP followed by a piecemeal CSP during the same session in a patient with extensive duodenal polyposis.

### Follow-up

Patients were evaluated 2 to 4 weeks after the procedure at the outpatient clinic or via a telephone consultation, to ensure that possible AEs were identified, discussed, and evaluated. Follow-up endoscopy was scheduled after 3 to 6 months when macroscopic resection was incomplete, high-grade dysplasia was present in the resected adenoma and/or when there were other lesions in the duodenum and/or stomach that required intervention. Otherwise, follow-up endoscopy was scheduled after 1 year. During follow-up endoscopy, the polypectomy scar(s) were assessed to detect recurrences.

### Outcome

The primary outcome was the occurrence of procedure-related AEs occurring within 30 days after CSP. AEs were evaluated using the validated Adverse Events in GI Endoscopy classification 24 . Deep mural injury was scored according to the Sydney classification system 25 .

### Statistical analysis

Descriptive statistics were used for this study. Continuous variables are presented as means with standard deviation for normally-distributed variables and as medians with interquartile ranges (IQRs) for skewed-distributed variables. Categorical variables are presented as numbers and percentages. All analyses were performed using SPSS 26 (IBM Corp. Released 2019. IBM SPSS Statistics for Windows, Version 26.0. Armonk, New York, United States: IBM Corp).

## Results

### Patient characteristics and included procedures

Thirty-nine consecutive FAP patients from three centers underwent CSP for a total of 133 superficial non-ampullary duodenal adenomas. During the same time period, 25 duodenal adenomas were resected with HSP. One patient underwent combined CSP and HSP of a large duodenal adenoma which, on histopathology, appeared to be an adenocarcinoma.

Patient characteristics are presented in Table 1 . The median age at (first) CSP was 47. Of the 39 patients, 22 (56%) had undergone an endoscopic duodenal polypectomy before entering the study. The total number of CSPs per patient in the study period varied from one to 22 with a maximum of 18 CSPs per session. No patients were on anticoagulants at time of CSP.

**Table:** **Table 1** Patient characteristics.

	39 FAP patients
Female sex, n (%)	26 (67%)
Proven *APC* mutation, n (%)	38 (97%)
Age at FAP diagnosis (median)	22 (IQR 17–29)
History of (procto)colectomy	35 (90%)
History of duodenal polypectomy	22 (56%)
Age at (first) CSP (median)	47 (IQR 37–56)
Number of CSP sessions	
One session	36 (92%)
Two sessions	3 (8%)
Number of CSPs (median)	2 (IQR 1–4, range 1–22)
Anticoagulants use at time of CSP	0
FAP, familial adenomatous polyposis; IQR, interquartile range; CSP, cold snare polypectomy.	

Table 2 summarizes the characteristics of the lesions and procedure outcomes. The median adenoma size was 10 mm (IQR 8–15). Fifty percent of the lesions were between 10 and 19 mm and 20% were ≥20 mm. Nearly all adenomas were located in D2 (69%) or D3 (26%) and had a flat elevated (IIa) (63%) or sessile (Is) (37%) appearance. Prior to CSP, most adenomas (82%) were submucosally lifted. Sixty-one lesions (46%) were resected en bloc and 72 (54%) piecemeal, resulting in a macroscopic radical resection rate of 97%. Most common difficulties during CSP were an unstable view (13%) and difficult location (5%). In 8% of lesions, hemoclips were prophylactically placed to prevent delayed bleeding or perforation.

**Table:** **Table 2** Adenoma characteristics and procedure outcomes in patients with FAP.

	CSP (n=133)
Size of adenoma (median, IQR)	10 (8–15)
5–9 mm	40 (30%)
10–19 mm	66 (50%)
20–39 mm	24 (18%)
40 mm	3 (2%)
Location of adenoma	
Bulb/D1	5 (4%)
D2	92 (69%)
D3	35 (26%)
D4	1 (1%)
Morphology (Paris classification)	
IIa	84 (63%)
Is	49 (37%)
Submucosal injection to lift lesion	109 (82%)
Adrenalin in submucosal injection	93 (70%)
Adjuvant snare tip soft coagulation	2 (2%)
En bloc resection	61 (46%)
Piecemeal resection	72 (54%)
Macroscopic radical resection	129 (97%)
Difficulties during CSP	
Difficult location	6 (5%)
Incomplete lifting	2 (2%)
Unstable frontal view	17 (13%)
Side-viewing endoscope needed	4 (3%)
Adverse events	
Intra-procedural bleeding	0
Perforation	0
Delayed bleeding	0
Deep mural injury type II	3 (2%)
Other adverse events	0
Prophylactic clip placement	10 (8%)
Histology	
Tubular adenoma	120 (90%)
Tubulovillous adenoma	13 (10%)
Grade of dysplasia	
Low-grade dysplasia	133
High-grade dysplasia	0
FAP, familial adenomatous polyposis; IQR, interquartile range; CSP, cold snare polypectomy.	

**Fig. 1:**
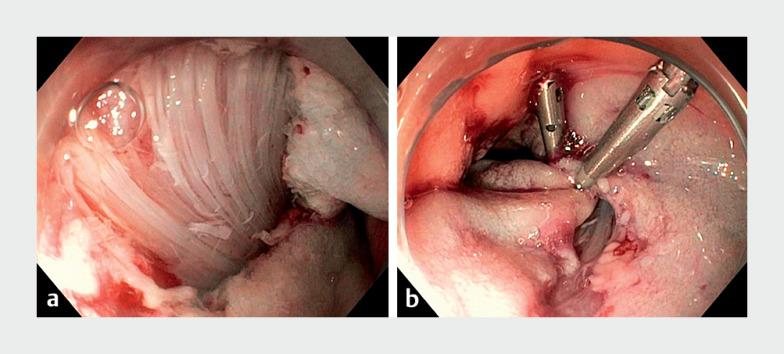
Deep mural injury type III with **a** damaged circular muscle layer, **b** prophylactically clipped.

In terms of intraprocedural AEs, no bleeding or perforation occurred. Three CSPs (2%) in three different patients resulted in full exposure of the muscularis propria, classified as deep mural injury type II (n=3). All three cases of muscularis propria exposure occurred after submucosal lifting and piecemeal resection for adenomas measuring 25, 20, and 45 mm, with the use of a dedicated cold snare. In two lesions there was a suspicion of submucosal scarring, which might have been caused by previous biopsies. All three resection sites were prophylactically closed with hemoclips to prevent delayed perforation, which did not occur ( Fig. 1 ). All three patients were admitted for observation.

After eight of 42 polypectomy sessions (19%), the patients were admitted for one or two nights due to either the resection of large adenomas (n=3) or a high number of resected adenomas (n=5). These admissions were planned and, therefore, not counted as AEs. Also, in the three cases of deep mural injury, the admission was had been planned prior to the procedure because of the number or size of the lesion(s). No delayed complications were observed either during the admission period nor the 4-week follow-up. Histology showed a tubular adenoma in 90% of lesions and tubulovillous adenoma in 10%. All lesions contained low-grade dysplasia; no high-grade dysplasia or cancer was detected.

## Discussion

In this multicenter study, we investigated the safety of CSP for the removal of superficial non-ampullary duodenal adenomas in FAP. We demonstrated that, in experienced hands, CSP is a safe technique, regardless of the size or number of adenomas resected during the same procedure.

Our series confirms the safety of CSP for duodenal adenomas because no bleeding or perforation was observed. Table 3 summarizes the available series on duodenal CSP in the sporadic and FAP setting showing the occurrence of intraprocedural bleeding (0%-90%), delayed bleeding (0%), and perforation (0%). These studies included CSPs with (cold EMR) and without submucosal lifting. Because the AE rate was low in all studies, we cannot state whether lifting results in fewer AEs. In the present study, most adenomas were lifted before CSP, and adenomas that were resected without lifting had a maximum size of 15 mm. We cannot draw any conclusions about larger lesions, but it might be hypothesized that performing CSP without lifting for larger adenomas might result in an increased risk of AEs. The only AE that occurred was deep mural injury type II during three procedures. In two of these lesions, there was a suggestion of submucosal scarring, which might have been caused by previous biopsies and might increase the risk of AEs and non-radical resection. Numbers are too small to further study this in the present series. In our current endoscopic surveillance protocol, we do not advise taking routine biopsies to prevent submucosal scarring 26 . Another factor that might increase the AE risk is the morphology of the adenoma. Resecting more bulky adenomas might result in a higher risk of bleeding, which is the reason to perform HSP for these adenomas in our current practice.

**Table:** **Table 3** Series on duodenal cold snare polypectomy.

Author, year	Setting	Design	N of CSPs	Adenoma size*	Intraprocedural bleeding	Delayed bleeding	Perforation
Choksi 2015 27	Sporadic	Retrospective	15	24 mm	0	1 (7%)	0
Maruoka 2017 21	Sporadic	Prospective	30	4 mm	27 (90%) ^†^	0	0
Hamada 2018 17	FAP	Prospective	332	NR	1 (3%)	0	0
Dang 2021 18	Sporadic	Retrospective	43	26.5 mm	0	0	0
Trivedi 2022 19	Sporadic	Retrospective	41	12 mm	4 (10%)	0	0
Repici 2022 20	Sporadic	Retrospective	33	31.5 mm	0	0	0
Wang 2023 22	Sporadic	Prospective	50	30 mm	1 (2%)	2 (4%)	2 (4%)
Present series	FAP	Prospective	133	10 mm	0	0	0
*Mean or median ^†^ Also bleedings that stopped spontaneously were counted as intraprocedural bleeding in this study CSP, cold snare polypectomy; FAP, familial adenomatous polyposis; NR, not reported.							

Two studies retrospectively compared CSP to HSP, showing that AEs occurred less frequently after CSP 19 20 , with intraprocedural bleeding occurring in 10% to 13% after HSP, delayed bleeding in 9% to 17%, and perforation in 1% to 10%. However, because these studies were both retrospective and might suffer from selection bias, one should be careful about drawing conclusions. No detailed information on lesion morphology was provided, and superficial lesions may have been resected using CSP and the bulkier lesions with HSP. Moreover, CSP has recently been introduced as a resection technique in the duodenum. Trivedi et al. showed that HSP was used for all but one lesion in polypectomies between 2006 and 2012, whereas most lesions were resected using CSP between 2018 and 2021. This timing is relevant due to the evolution of hemostatic techniques, which were not available or widely used in the early years of duodenal polypectomy for FAP. Therefore, it is difficult to compare bleeding rates between the two techniques. Besides, the resection technique will be based on polyp morphology, introducing another bias. A randomized trial would eliminate most of these biases, but it is questionable whether a randomized trial is necessary or ethically justifiable, with such positive current data on CSP.

In terms of efficacy, evaluating adenoma recurrences after duodenal polypectomy might be of importance. In this study, we did not report on recurrences after CSP. FAP patients undergoing duodenal polypectomy usually have numerous adenomas and sometimes multiple adenomas are removed during the same procedure. We believe that this clinical setting does not lend itself to studying adenoma recurrence, because this is clinically less relevant, given that patients usually have multiple duodenal adenomas and should undergo regular surveillance endoscopies anyway. This is in contrast to the sporadic setting, in which usually one polyp is removed in an otherwise normal duodenum and surveillance might be terminated after complete removal. Two studies in the sporadic setting comparing CSP to HSP did not find a difference in recurrence rate 19 20 . Polyp size was found to be a predictor of recurrence in univariate analysis 19 . Removing adenomas ≥5 mm rather than ≥10 mm as recommended by current guidelines might result in fewer recurrences and more en bloc resections. It could also prevent unmanageable duodenal disease with multiple large adenomas, which may require more complex polypectomies in the future. Takeuchi et al. 11 introduced a different endoscopic approach, called intensive downstaging polypectomy for duodenal polyposis in FAP, in which all large and small duodenal adenomas are resected, mostly using CSP. It resulted in downstaging of duodenal polyposis in most patients (71%). However, whether removing adenomas <5 mm results in a lower risk of developing duodenal cancer is debatable. This intensive strategy with frequent endoscopies including a high number of polypectomies results in a burden for the patients and potentially also an increased complication risk. Although future studies will have to guide the trade-off between safety and efficacy of CSP in terms of recurrence and feasibility, taking into account lesion size and morphology, we believe CSP is the preferred method for resecting (multiple) superficial duodenal adenomas in FAP. Whereas in the past data from retrospective studies were used for counseling, reporting on different resection techniques combined, we are now able to more accurately inform patients about risks before they undergo CSP.

## Conclusions

We demonstrated the safety of CSP in FAP patients, but not its efficacy in preventing cancer and surgery. Long-term prospective data from a large FAP cohort are needed to evaluate whether protocolized removal of duodenal adenomas is an effective strategy and, more specifically, which adenomas should be removed. As a European consortium, we hope to provide such data in future years.
